# Evaluation of the immunotoxicity and allergenicity of a new intranasal influenza vector vaccine against tuberculosis carrying TB10.4 and HspX antigens

**DOI:** 10.22038/IJBMS.2023.68440.14936

**Published:** 2023

**Authors:** Kira I. Stosman, Andrey G. Aleksandrov, Konstantin V. Sivak, Zhanna V. Buzitskaya, Marina A. Stukova

**Affiliations:** 1 Smorodintsev Research Institute of Influenza, 197376, Ulitsa Professora Popova 15/17, St. Petersburg, Russian Federation

**Keywords:** Guinea pigs, Immunity, Mice, Tuberculosis, Vaccine

## Abstract

**Objective(s):**

A new vaccine candidate TB/FLU-05E has been developed at the Smorodintsev Research Institute of Influenza (Russia). The vaccine is based on the attenuated influenza strain A/PR8/NS124-TB10.4-2A-HspX that expresses mycobacterial antigens TB10.4 and HspX. This article describes the results of preclinical immunotoxicity and allergenicity studies of the new vector vaccine TB/FLU-05E against tuberculosis.

**Materials and Methods:**

The experiments were conducted on male CBA mice, С57/black/6 mice, and guinea pigs. The vaccine candidate was administered intranasally (7.7 lg TCID_50_/animal and 8.0 lg TCID_50_/animal) twice at a 21-day interval. The immunotoxic properties of the vaccine were assessed in mice according to the following parameters: spleen and thymus weight and their organ-to-body weight ratio, splenic and thymic cellularity, hemagglutination titer assay, delayed-type hypersensitivity test, and phagocytic activity of peritoneal macrophages. Histological examination of the thymus and spleen and white blood cell counts were also performed. Allergenicity of the vaccine was assessed in guinea pigs using conjunctival and general anaphylaxis reaction tests.

**Results:**

The results showed that double immunization with the TB/FLU-05E vaccine did not affect the phagocytic activity of peritoneal macrophages, cellular and humoral immunity after immunization with a heterologous antigen (sheep red blood cells), or the organ-to-body weight ratio of immunocompetent organs (thymus and spleen). The vaccine candidate demonstrated no allergenic properties.

**Conclusion:**

According to the results of this study, the TB/FLU-05E vaccine is well-tolerated by the immune system and demonstrates no immunotoxicity or allergenicity.

## Introduction

Tuberculosis (TB) is an infectious disease and one of the ten most common causes of death in the world. Large numbers of new infections and the threat of multidrug-resistant and extensively drug-resistant strains of *Mycobacterium tuberculosis* (MTB) necessitate the development of a new TB vaccine (1). Currently, there are different types of vaccines in the TB vaccine pipeline, including live attenuated, inactive, and subunit vaccine candidates (2, 3). Considering the natural route of TB infection, mucosal administration is actively employed by many researchers since it has the potential to provide physiological and immunological advantages against MTB infection (4).

Antigens and adjuvants used in vaccines vary in their mode of action and ability to stimulate the immune system (5). Evidence suggests that several mechanisms which are responsible for the immunostimulatory effects are also responsible for the adverse effects of vaccines (6). As with any medication or biological product, people can be allergic to vaccines. Vaccine anaphylaxis is rare and occurs primarily in individuals with a history of allergies to the components of the vaccines, such as antigens, adjuvants, stabilizers, preservatives, emulsifiers, cell culture materials, inactivating ingredients, etc. Vaccines cultured in developing chicken embryos contain a small amount of the ovalbumin protein which can also provoke allergic reactions. Allergic reactions include swelling with itching at the injection site, conjunctivitis, rhinorrhea, bronchoconstriction, generalized urticaria, bronchospasm, and life-threatening anaphylaxis (7). Preclinical evaluation of immunotoxicity and hypersensitivity reaction allows researchers to identify adverse events that may develop after vaccination. Immunotoxicity and allergenicity studies of the vaccines are necessary stages in the research and development of immunobiological medical products for clinical usage in Russia. 

The basic principles of a nonclinical study of vaccines are presented in the European Medicines Agency (EMA) guidelines, the World Health Organization (WHO) guidelines, the United States Food and Drug Administration (FDA) guidelines, as well as in national compendia (Russian, Chinese, Japanese guidance documents, etc.). The EMA guideline and the FDA guideline outline similar principles for evaluating the safety and efficacy of therapeutic products. At the same time, EMA and FDA also recognize The International Council for Harmonization (ICH) guidelines. ICH has issued safety guidelines to identify potential risks, including the immunotoxicity risk.

Toxicity to the immune system includes many effects such as suppression or activation of the immune response. Suppression can lead to a decrease in the host’s resistance to infections or the appearance of tumor cells; activation can aggravate autoimmune diseases or induce an allergy.

Evaluation of vaccine immunotoxicity risk includes testing for immunogenicity. This assessment is based on quantitative and qualitative changes which characterize humoral, cell-mediated, and innate immune responses (8, 9). The study of protection and immunogenicity is recommended to be performed on a relevant animal model (10). Immunotoxicity testing requires scientific flexibility. Routine research methods for new pharmaceuticals are not recommended for vector vaccines (10).

A new intranasal candidate vaccine against tuberculosis TB/FLU-05E was developed at the Smorodintsev Research Institute of Influenza (Russia). It is a replication-deficient attenuated mucosal vector vaccine generated from the influenza virus that expresses TB10.4 and HspX Mtb antigens (Flu/THSP vaccine virus). 

There are quite a lot of studies on the safety and immunogenicity of vaccines, but studies of the immunotoxic potential of these vaccines are scarce. Here we present the results of pre-clinical immunotoxicity and allergenicity studies of the new TB/FLU-05E vector vaccine against TB.

## Materials and Methods

***The TB vaccine candidate***

The recombinant vector vaccine for TB prevention TB/FLU-05E was developed at the Smorodintsev Research Institute of Influenza (Russia) based on the attenuated influenza strain A/PR8/NS124-TB10.4-2A-HspX expressing mycobacterial antigens TB10.4 and HspX (Flu/THSP). The design of the virus and vector are described in detail by Sergeeva *et al*. (11). The vaccine candidate was produced in chicken eggs. The harvest was purified by consequent clarification, concentration, and diafiltration and formulated in a sucrose-phosphate-glutamate stabilizing buffer (SPGN) with 5 mg/dose of Recombumin (Albumedix Ltd., UK). The stabilizing buffer was used as a control in the animal studies (Placebo). The vector vaccine candidate TB/FLU-05E was administered in single and double immunization doses (ID). One ID equals 7.7 lg Tissue Culture Infectious Dose (TCID_50_) and 2 ID corresponds to 8.0 lg TCID_50_.

***Laboratory animals***

Animal experiments were performed according to European and national directives for the protection of experimental animals and were approved by the Bioethics Committee at the Smorodintsev Research Institute of Influenza (Ethic №77 dated 05 June 2019). The research was conducted on 150 CBA mice (males weighing 18–20 g), 60 С57/black/6 mice (males weighing 18–20 g), and 30 guinea pigs (males weighing 300–350 g). Animals were obtained from an accredited laboratory animal nursery of the “Stolbovaya” branch of the Federal State Budgetary Institution of Science “Scientific center for biomedical technologies of the Federal Medical and Biological Agency” (Moscow, Russian Federation). 

***Experimental design***

Animals were randomly divided into 3 groups. The first group (n=10) was treated with the control buffer (SPGN). The second group (n=10) was immunized with the TB/FLU-05E vaccine at a dose of 7.7 lg TCID_50_ (1 ID). The third group (n=10) was immunized with the vaccine at a dose of 8.0 lg TCID_50_ (2 ID). Mice and guinea pigs were immunized twice at a 21-day interval via the intranasal route. The study of the general anaphylactic reaction was carried out in guinea pigs following another protocol. The immunization schedules are described below.

***Evaluation of hematological parameters in CBA mice***

Blood was collected from the retro-orbital plexus of each mouse before sacrificing it by cervical dislocation. The blood was collected into sterile EDTA anticoagulant tubes for white blood cell count (WBC) analysis. Blood smears were also prepared, stained with Giemsa dye, and then differentially analyzed with a light microscope (based on cell counts of at least 200 cells per slide/mouse).

***Measurement of splenic and thymic weight and cellularity***

Mice lymphoid organs (thymus and spleen) were weighed; cell suspension in the 199 medium was prepared using a glass homogenizer. The suspension was filtered through nylon filters, washed twice, and stained with azure-eosin. Nucleated cells were counted microscopically (12).

***Evaluation of humoral immunity using hemagglutination (HA) titer assay in CBA and С57/black/6*** ***mice ***

The effect of the vaccine on the inductive and productive phases of antibody synthesis was evaluated after immunization with sheep red blood cells (SRBC, Moscow, Russian Federation). Mice received the antigen (5×10^6^ SRBC) after the second vaccine administration to assess the inductive phase; the antigen was administered on the third day after the second vaccine immunization to assess the production phase. Blood serum was obtained eight days after SRBC immunization to determine the titer of hemagglutinins. 25 μl aliquots of two-fold serum dilutions in PBS were combined with 25 μl of a 2% [v/v] SRBC suspension in microplate wells. The microplate was incubated at 37 ºC for 1 hr and checked for hemagglutination (button formation). The highest dilution that promoted hemagglutination was taken as the antibody titer. The mean titer was expressed as log_2 _(13, 14).

***Evaluation of cellular immunity in a delayed-type hypersensitivity reaction in CBA mice***

Mice were sensitized by subcutaneous injection of 2×10^8^ SRBCs diluted in 100 μl of saline. They were challenged with a 50 μl booster dose of 10^8^ SRBCs in the left hind footpad on day 5 after sensitization. The right hind footpad was injected with the same volume of PBS for trauma control for non-specific swelling. The local inflammatory response was measured 24 hr after the SRBC challenge (12). The mean increase in footpad weight was calculated as 100% × (Left footpad [challenged with SRBC] weight – Right footpad weight) / Right footpad weight).

***Phagocytic activity of peritoneal macrophages in CBA mice***

Briefly, 100 μ1 aliquots of each sample were pipetted into microplate wells. 10 μ1 of 0.33% neutral red solution were added to each well and samples were incubated for 1 hr at 10 °C. The cells were centrifuged at 200 g for 5 min and washed twice in a buffer. 100 μ1 aliquots of l % acetic acid in 50% ethanol were added to all wells (15). The plates were incubated for 15 min at 20 °C and were read at 550 nm in a spectrophotometer Epoch 2 (BioTek Instruments, USA). 

***Histological examination of spleen and thymus***

Thymus and spleen were isolated from mice after double vaccine immunization. The tissue was fixed in 10% formaldehyde, washed, dehydrated, and embedded in paraffin blocks. Standard sample processing was carried out using a Histo-TekVP1 automated tissue processor. Paraffin blocks were cut into 5 µm tissue sections and stained with hematoxylin and eosin. The histological sections were examined using a Leica DM1000 microscope.

***General anaphylaxis in guinea pigs***

For sensitization, guinea pigs were subcutaneously and intramuscularly injected with the vaccine or SPGN three times. The challenge intracardiac dose equal to the total sensitizing dose was administered on day 14 after the sensitization. The same dose was administered to guinea pigs in the control group. All immediate allergic reactions occurring within 30 min after intracardiac injection were observed and graded based on symptoms (12). 

***Conjunctival test*** ***in guinea pigs ***

A conjunctival test was performed in guinea pigs on day 10 after two intranasal administrations of the TB/FLU-05E vaccine or placebo. 0.05 ml of the vaccine was applied under the upper eyelid in all animals. An equal amount of distilled water was applied under another upper eyelid as a control. The reaction was monitored for 15 min and then assessed 24 hr after the test. The state of the sclera, cornea, and eyelids was evaluated (12).

***Statistical analyses***

The Prizm 8.0 software (GraphPad Software, Inc., USA) was employed for statistical analyses. Data were tested for normality using the Shapiro–Wilk test. Differences between groups of normally distributed data were assessed using one-way ANOVA. Mann-Whitney U-test or Kruskal–Wallis test was applied to non-normally distributed data, followed by Dunn’s *post hoc* analysis. *P*-values of 0.05 or less were considered significant. Values are presented as Mean±SEM.

## Results

***Immunotoxicity***

***Hematological study***

No statistically significant differences in WBC analysis were found between the experimental group of mice after the vaccine administration and the control (Table 1). 

***Measurement of splenic and thymic weight and cellularity***

**Figure 1:**
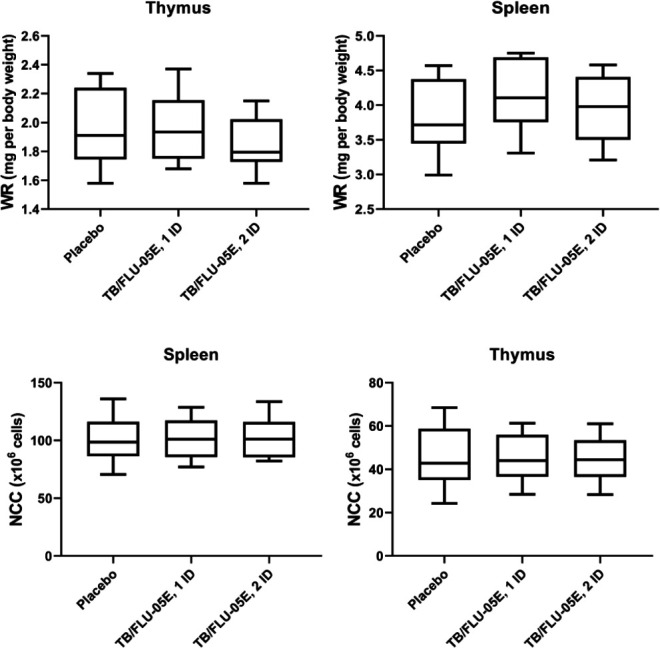
Thymus and spleen weight ratios (organ, mg per body weight, g), the number of nucleus-containing cells (×10^6^ cells) in immunized CBA mice WR: weight ratios, NCC: Nucleus-Containing Cells, ID: immunization doses. Data are shown as Mean±SEM, n= 10

No treatment-related macroscopical findings or changes in organ weight and organ-to-body weight ratio values were evident in any of the vaccine-treated animals after the second vaccination. Macroscopically, the thymus and spleen in the experimental group did not differ from the control group. The thymus was triangular, whitish in color, and slightly dense in texture. The surface of the spleen had a dark cherry color, was smooth and grayish, and small-cell follicles were visible on the slice. No changes in splenic and thymic cellularity were observed in mice (Figure 1). 

***Hemagglutination (HA) titer assay***

Two different lines of mice, CBA and C57BL/6, were used to study the impact of the vaccine on humoral immunity. Possible immunotropic effects were evaluated in the inductive and productive phases of antibody generation. The administration of the vaccine led to neither stimulation nor suppression of the humoral immune response. Titers of antibodies produced in response to SRBC illustrate that the vaccine did not affect the formation of antibodies (Table 2). 

***Delayed-type hypersensitivity*** *.*

The effect of the TB/FLU-05E vector on cell-mediated immune response was studied using the delayed-type hypersensitivity (DTH) test induced by SRBC. The results show that the vaccine did not stimulate or inhibit the DTH reaction in mice, which indicates that the vaccine does not negatively affect cell-mediated immunity. After vaccination, mice demonstrated no adverse events during the 24-hr DTH response. The presented results suggest that the vaccine does not affect the hypersensitivity reaction to SRBC (Table 3). 

***Phagocytic activity of peritoneal macrophages***

The test for differential uptake of neutral red by peritoneal macrophages detected no differences in the functional activity of immunocompetent cells between immunized and control animals (Table 4).

***Histological examination of spleen and thymus***

Histological examination after the TB/FLU-05E immunization revealed clearly delineated thymic lobules with prominent cortex and medulla. The cortex of the lobules was filled with lymphocytes and thymocytes. The medulla contained fewer lymphocytes. No organ atrophy after immunization was observed. A clear distinction between the red and white pulp, resting follicles, and marginal zones was evident in the spleen of experimental and control mice. Normal histological structure of the spleen was preserved after vaccination. Follicles demonstrated no hyperplasia, and lymphoreticular elements of the spleen had clear nuclei. No tumors were detected. No differences in the cortical and medullar layers between any experimental groups were found.

***Allergenicity***

***General anaphylactic reaction***

The administration of the challenge vaccine dose did not trigger any allergic reactions in sensitized animals. No rapid breathing or scratching of the body and muzzle with hind legs was observed. Convulsions, asphyxia, bronchospasm, and lethality were absent. No differences between any experimental groups were found.

***Conjunctival test***

There was no reaction to the vaccine administration during a conjunctival test in guinea pigs during either period of observation (for 15 min and then 24 hr after the test). Reddening of the lacrimal duct, sclera, or conjunctiva was not recorded. No differences between any experimental groups were found.

**Table 1:** Hematological profile of mice after TB/FLU-05E immunization

Parameters	Placebo	TB/FLU-05E, 1 ID	TB/FLU-05E, 2 ID
WBC (10^9^/L)	7.00±0.71	8.64±0.39	9.11±0.30
Basophils (10^9^/L)	0.05±0.02	0.06±0.02	0.07±0.02
Neutrophils (10^9^/L)	1.66±0.2	2.02±0.31	1.49±0.03
Monocytes (10^9^/L)	0.09±0.05	0.17±0.06	0.13±0.05
Lymphocytes (10^9^/L)	5.17±0.71	5.97±0.08	7.32±0.22

Data are given as mean±SEM for n=10

**Table 2.** Antibody generation in response to sheep red blood cells (SRBC) after TB/FLU-05E immunization

Parameters	Placebo	TB/FLU-05E, 1 ID	TB/FLU-05E, 2 ID
	***СВА*** ***mice***		
IgG titer, Log_2 _(IPh)	5.4±0.2	***6.1±0.4***	***6.2±0.4***
IgM titer, Log_2 _(IPh)	2.3±0.2	***2.7±0.2***	***2.1±0.2***
IgG titer, Log_2_(PPh)	4.7±0.4	***4.4±0.3***	***4.3±0.4***
IgM titer, Log_2 _(PPh)	2.3±0.2	***2.2±0.1***	***2.3±0.2***
	С57ВL/6 mice		
IgG titer, Log_2 _(IPh)	5.5±0.4	***5.6±0.5***	***4.2±0.5***
IgM titer, Log_2 _(IPh)	2.4±0.2	***2.3±0.2***	***2.2±0.1***
IgG titer, Log_2_(PPh)	4.5±0.5	***4.9±0.3***	***4.4±0.4***
IgM titer, Log_2 _(PPh)	2.2±0.1	***2.3±0.2***	***2.1±0.1***

Data are presented as mean±SEM for n=10. IPh: Inductive phases of antibody generation, PPh: Productive phases of antibody generation. Ig: immunoglobulin, ID: immunization doses – please, check a fonts

**Table 3:** Effect of TB/FLU-05E immunization on the delayed-type hypersensitivity reaction in mice

Treatment	Parameters		
	Weight of the experimental paw, mg	Weight of the control paw, mg	Index of the reaction
Placebo	190.80±3.76	114.50±2.10	66.99±3.79
TB/FLU-05E, 1 ID	202.80±4.58	119.50±1.67	69.97±4.36
TB/FLU-05E, 2 ID	199.70±2.57	112.70±2.18	77.79±4.18

ID: immunization doses. Data are presented as mean±SEM for n=10

**Table 4:** Influence of TB/FLU-05E immunization on the phagocytic activity of peritoneal macrophages in mice

Parameters	Treatment		
	Placebo	TB/FLU-05E, 1 ID	TB/FLU-05E, 2 ID
Phagocytic activity, o.d.	0.35±0.03	0.37±0.03	0.39±0.03

ID: immunization doses. Data are presented as mean±SEM for n=10. o.d.: optical density

## Discussion

TB remains a global health problem. Numerous new candidate vaccines are therefore being developed and tested worldwide. Preclinical trials aim to assess the potential toxicity of a new vaccine candidate in animals before it can enter clinical trials in human participants. 

The ICH guidelines highlight the need to identify hematological changes, changes in the immune system, weight changes in the thymus, spleen, and other organs; changes in serum globulins; increased incidence of infections; increased occurrence of tumors; and histological examination of immunocompetent organs during studying the safety of new pharmaceuticals (16). At the same time, one essential aspect of the immunotoxicological evaluation of biotechnology-derived pharmaceuticals is assessing their potential immunogenicity (9).

Previously, the results of the evaluation of TB/FLU-05E immunogenicity were presented as recommended by most guidelines (ICH, WHO, etc.). It was shown that the mucosal TB vaccine vector candidate induced the incoming of interstitial macrophages in the lung tissue and enhanced T-cellular immune response, which was mediated by antigen-specific effector and central memory CD4+ and CD8+ T-lymphocytes (11, 17). The Flu/THSP vector was safe and stimulated a systemic TB-specific CD4+ and CD8+ T-cell immune response after intranasal immunization in mice. The vaccine protected mice from severe lung injury caused by MTB infection. 

While evaluating the protection and immunogenicity of TB/FLU-05E, we also paid attention to the potential immunotoxicity of the vaccine candidate. Changes in these parameters could reflect immunosuppression or activation.

WHO recommends studying the nonspecific effects of vaccines on unrelated microbial stimuli (10, 18). Several vaccines are known to alter antibody responses to unrelated antigens suggesting an effect on B-cell function (19). For example, Bacillus Calmette–Guérin (BCG) vaccination increases heterologous responses to poliovirus vaccination (20), to anti–pneumococcus, anti–hemophilus type B, and anti–tetanus toxoid vaccines (21), as well as to hepatitis B vaccine (22). It is also shown to boost anti-A(H1N1)pdm09 antibody titer levels (23). On the other hand, no influence on the humoral immune response was found while investigating the immunomodulatory effects of BCG vaccination on antibody responses to heterologous vaccines/antigens. Administration of *Diphtheria toxoid* (DT) had no influence on anti-DT antibody titer levels in BCG-vaccinated infants; vaccination against hepatitis B (HepB) did not change anti-HepB antibody titer levels in BCG-vaccinated subjects (24).

We also studied the influence of TB/FLU-05E on antibody responses to heterologous antigens in mice. Mice immunized with the heterologous T-dependent antigen (SRBC) did not demonstrate any change in serum antiSRBC–antibody titer (IgG and IgM) in either the inductive or productive phases of antibody synthesis. The results obtained allow us to conclude that there is no suppression of the humoral immune response, which suggests that vaccinated people would respond adequately to any heterologous infectious agent.

Vaccines are immunomodulating pharmaceuticals and can be expected to trigger a cellular immune response. For example, BCG is known to affect the innate and T cell-mediated immune responses to proteins, peptides, or conjugated polysaccharide vaccines, which behave as T-dependent antigens (23, 25). Heterologous effects of BCG on T-cell immunity were observed in elevated IL-22 levels in response to *E. coli* and *C. albicans* (26). A single dose of the MTBVAC vaccine (the subcutaneous route with MTBVAC 10^6^ CFU) induced an enhanced proinflammatory response against heterologous stimulation (10 μg/mice of LPS was administered by intraperitoneal injection) four weeks after vaccination in mice (27). 

We evaluated the effect of TB/FLU-05E on the cellular immune response to a heterologous antigen (SRBC). Evaluation of DTH is a useful approach for assessing cell-mediated immune responses that are associated with Th1 reactivity (28). The DTH reaction is mediated by CD4+ T-lymphocytes, which promote T-helper cell type 1 (Th1) production of interferon-γ. The DTH response can be evaluated by monitoring localized swelling, leukocyte infiltration of the challenged tissues, and Th1-associated cytokine profiling. The absence of Th1 reactivity-dependent cell-mediated immune response suggested the adequate DTH reaction in our experiment. This fact allows us to conclude that the cellular immune system does not react negatively to vaccination with TB/FLU-05E in mice.

In addition to specific induction of lymphocyte responses, vaccines can modulate nonspecific innate immune responses (29, 30). For example, a decrease in the level of anti-inflammatory cytokine IL-10 during stimulation with LPS implies that BCG shifts the balance towards a more pro-inflammatory response to LPS (26). BCG vaccination leads to increased cytokine production in response to non-related pathogens for up to three months after vaccination in healthy volunteers (31). We evaluated the phagocytic activity of peritoneal macrophages to identify possible adverse effects of TB/FLU-05E on innate immunity. We observed no changes in the functional activity of innate immunity in mice.

Changes in spleen and thymus weight and their cell counts often reflect xenobiotic-induced alterations in the immune system. Reduced cell numbers in these immunocompetent organs may indicate a direct cytolytic action of the xenobiotic on lymphocytes. An increase in spleen cell number often results from a proliferative response to the xenobiotic. Increased thymic cell size indicates the activity of T-cell lymphopoiesis and cytokine production. The thymus is the main lymphoid organ that regulates the immune and endocrine systems by controlling thymic cell proliferation and differentiation. The hematological profile provides important information about the host’s response to trauma, deprivation, and/or stress, as well as inflammation (32). Therefore, the hematological profile was evaluated in our study of the immunological properties of the TB/FLU-05E vaccine.

Double immunization affected neither the absolute leukocyte level nor splenic and thymic weight and cellularity. We observed no signs of immunosuppression, such as depletion or hyperplasia in the splenic white pulp or changes in cortical (T-cell) and medullar (B-cell) areas. Normal thymus architecture was also preserved after vaccination. Hence, our study showed no suppression of the immune response, which could lead to a decrease in the host’s resistance to infections or the appearance of tumor cells. 

Adverse effects after administration of vaccines, such as drug allergy, are frequently reported in the general population. An example of a potentially life-threatening allergic reaction to foreign antigens is anaphylaxis. This type 1 immediate hypersensitivity reaction is mediated by immunoglobulin E (IgE) and usually occurs within minutes to hours after injection. BCG, the most widely used vaccine for the prevention of tuberculosis worldwide, is a potent inducer of the Th1 response. Despite this, side effects of the BCG vaccine, such as anaphylactic reactions, are very rare. BCG revaccination does not modulate total IgE levels in vaccine responders or allergen-specific IgE levels in the study population (33).

The EMA Guideline on quality, non-clinical and clinical aspects of live recombinant viral vectored vaccines attributes little importance to the routine evaluation for allergic reactions of these types of products (10). However, the Russian national guideline (12) recommends evaluating the ability of candidate vaccines to induce hypersensitivity reactions or individual sensitivity reactions in animals. In our work, special attention was given to immediate and delayed hypersensitivity in animals after vaccination. The study of the allergenic response to the vaccine showed no anaphylactic reaction (type I hypersensitivity) or DTH (type IV) in sensitized guinea pigs. 

To sum up, the results of our study suggest that the TB/FLU-05E vaccine has no immunotoxic potential and induces no allergic reactions. Additionally, we have previously shown that the ТВ/FLU-04L vaccine using a vector similar to TB/FLU-05E induced no significant changes in the metabolism or hematopoiesis indicators, and no morphological changes in the organs were identified (34, 35). Degenerative processes, changes associated with necrobiosis, or inflammation of experimental animals’ inner organs were not observed. No tumors were detected in any studied organs during visual and micro-examination.

According to the studied parameters, it can be concluded that the new TB/FLU-05E vaccine is a safe drug and it could contribute significantly to the fight against tuberculosis infection after passing further clinical studies.

## Conclusion

The TB/FLU-05E vaccine, administered in doses of 7.7 lg TCID_50_ and 8.0 lg TCID_50_, demonstrated no immunotoxicity or allergenicity in preclinical studies conducted on two animal species (mice and guinea pigs). There were no mortalities or noticeable systemic or local clinical signs of allergy in vaccinated animals, and no significant changes in the analyzed parameters of the immune system were observed. Summing up the results of the three used tests, we conclude that the TB/FLU-05E vaccine candidate is safe in terms of its allergenic properties and immunotoxicity.

## Authors’ Contribution

KVS, ZhB, and MS designed the experiments; KIS and AA performed experiments and collected data. KVS and MS supervised the research. KIS, AA, KVS, ZhB, and MS analyzed the data. KIS prepared the original draft. AA and ZhB helped with writing and editing.

## Conflicts of Interest

The authors of this study do not have any conflicts of interest to declare.
